# The Effect of Changing Weekly Contact Training Duration Beyond Current Guidelines on Head Acceleration Events in Rugby Union

**DOI:** 10.1007/s40279-025-02359-3

**Published:** 2025-11-27

**Authors:** Thomas Sawczuk, Greg Roe, James Tooby, Cameron Owen, James Brown, Matt Cross, Éanna Falvey, Mark S. Gilthorpe, Sharief Hendricks, Samuel Hudson, Simon Kemp, Lindsay Starling, Keith Stokes, Ross Tucker, Ben Jones

**Affiliations:** 1 Carnegie Applied Rugby Research (CARR) Centre, Carnegie School of Sport, Leeds Beckett University, Leeds, UK; 2 Obesity Institute, Leeds Beckett University, Leeds, UK; 3 England Performance Unit, Rugby Football League, Manchester, UK; 4 World Rugby, Dublin, Ireland; 5 Division of Physiological Sciences, Department of Human Biology, Faculty of Health Sciences, University of Cape Town, Cape Town, South Africa; 6 Department of Exercise, Institute of Sport and Exercise Medicine (ISEM), University of Stellenbosch, Stellenbosch, South Africa; 7 PREM Rugby, London, UK; 8 School of Medicine and Health, University College Cork, Cork, Ireland; 9 Centre for Health and Injury and Illness Prevention in Sport, University of Bath, Bath, UK; 10 UK Collaborating Centre On Injury and Illness Prevention in Sport (UKCCIIS), University of Bath, Bath, UK; 11 Rugby Football Union, Twickenham, UK; 12 London School of Hygiene and Tropical Medicine, London, UK; 13 Faculty of Health Sciences, School of Behavioural and Health Sciences, Australian Catholic University, Brisbane, QLD Australia

## Abstract

**Background:**

This study simulated the effect of reducing contact training duration on overall in-season head acceleration event (HAE) exposure within men’s and women’s rugby union.

**Methods:**

Players (*n* = 982) from two professional men’s and two semi-professional women’s competitions wore instrumented mouthguards in training and match-play for one season. Generalised linear mixed models were used to estimate the in-season weekly HAE exposures per position, sex and contact type. Simulation of modelled estimates evaluated the impact of reducing contact load guidelines by 25%, 50% and 75% (scenario 1), and replacing full contact training with controlled contact (scenario 2) or non-contact (scenario 3) training for different seasonal match exposures. Previously established contact load guidelines were used as a reference point.

**Results:**

HAEs were decreased by a maximum of 3.2 per week (0–95 HAEs per season; 0–23%). In scenario 1, the decrease in HAEs was disproportionately smaller than the reduction in contact training duration (e.g. 23.7% reduction in overall rugby minutes for 7% decrease in HAEs). Scenario 2 decreased HAEs similarly to scenario 1 but with no reduction in contact time. Scenario 3 decreased HAEs proportionally with contact time reductions (e.g. 8.9% decrease in HAEs >10 *g* for 9.6% reduction in overall rugby minutes).

**Conclusions:**

HAEs were reduced in all scenarios, but the reduction was relatively small due to the low overall rate of HAEs in training. Policymakers should be aware of the tradeoffs involved in any change. Managing individuals with higher HAE exposures may be more appropriate than reducing contact training guidelines.

**Supplementary Information:**

The online version contains supplementary material available at 10.1007/s40279-025-02359-3.

## Key Points

**Table:** 

This study introduces simulation as a method of evaluating reductions in contact training duration beyond current guidelines in rugby without having to collect longitudinal data for every possible scenario.
Reducing weekly contact training durations across all contact types would decrease cumulative HAE exposure for all players by a maximum of 3.2 HAEs per week, but would require a disproportionately large reduction in contact training duration.
Replacing full contact training with controlled contact or non-contact training is at least as effective as reducing all contact training time by 50% but would result in players not experiencing full contact rugby outside of match-play, thus could only feasibly be implemented in players who frequently participate in matches and have high HAE exposure.

## Introduction

Within contact sports, such as rugby union, there is growing interest in the long-term impact of head acceleration events (HAEs) on brain health [1, 2]. It has recently been suggested that the cumulative effect of HAEs may have a greater association with long-term health outcomes than the number of concussions a player experiences [3, 4]. Consequently, policymakers in some contact sports (e.g. rugby league [5]) have introduced player match exposure guidelines (i.e. the number of minutes they can play within a predefined time period) to reduce their HAE exposure, whilst other sports (e.g. rugby union) have implemented match limits on the basis of injury risk and player load principles [6–8]. In addition to match exposure limits, policymakers may wish to explore other avenues of reducing HAEs, such as managing training exposure, as has previously been considered within American football [9].

Elite rugby players participate in multiple training sessions per week. Alongside matches, these sessions could contribute to career HAE exposure, but are necessary to prepare for match-play and develop contact technique, optimising performance and reducing the risk of match injuries [10]. Contact load guidelines have been provided by World Rugby to manage this tradeoff [11]. These guidelines, produced by expert consensus, suggest that players should participate in no more than 15 minutes full contact, 40 minutes controlled contact and 30 minutes of set piece training per week. However, no study has quantitatively considered whether or how changing these suggested limits could decrease HAE exposure in rugby.

Evaluating whether, and to what extent, changing contact load training time could decrease in-season HAE exposure is complex. Challenges include contextualising HAEs for players when there is wide variation in the number of matches players play [5]; week-to-week variations in the quantity and intensity of contact training undertaken by teams and players [11]; and varying usage of instrumented mouthguards (iMG) by players during training [12]. Together, these challenges have resulted in only small samples of training HAE data being collected to date [13, 14], which has limited the insights that can be gained.

One method through which greater insights can be obtained from small samples of data is simulation. Simulation studies are experiments which create data by pseudo-random sampling from known probability distributions [15, 16]. In the context of HAEs in contact sports, simulation studies allow researchers to create synthetic populations of athletes using the results of statistical models from samples of previously collected data. Extrapolating the results of these models through simulation studies is particularly advantageous to policymakers and practitioners alike as it allows different scenarios (e.g. differing number of matches played and/or policy changes) to be evaluated, without requiring the collection of large quantities of longitudinal data for each individual scenario. Therefore, the aim of this study was to use simulation techniques to evaluate the effect of changing weekly contact durations relative to the current World Rugby contact load guidance [11] on overall in-season HAE exposure, across a range of training reductions and match exposures.

## Methods

### Study Design

This study was completed in two parts. First, iMG data from training and match-play in men’s and women’s rugby union were used to model the incidence of HAEs per minute of training, split by contact type and match-play. Second, simulation of these modelled estimates evaluated the effect of changing weekly contact duration relative to World Rugby contact load guidelines on players’ overall in-season HAE exposure across a range of training reductions and match exposures. Three scenarios were considered: scenario 1: reduction in weekly contact training duration (i.e. full contact, controlled contact and set piece training) by 25%, 50% or 75% and replaced with non-contact training; scenario 2: replacing full-contact training with controlled-contact training; and scenario 3: replacing full-contact training with non-contact training.

The dataset was obtained from a prospective observational study in rugby union players from two professional men’s and two semi-professional women’s rugby union competitions [13]. The highest levels of domestic competition were included. Men’s rugby data came from England (Premiership Rugby) and South Africa (Currie Cup); women’s rugby data came from England (Premiership Women’s Rugby) and New Zealand (Farah Palmer Cup). A total of 982 individual players wore iMGs during training (1456 in-season player training weeks) and match-play (4152 player matches) across a combined total of 132 unique training weeks and 365 unique matches from one in-season period. Ethics approval was received from the Leeds Beckett University Ethics Committee (REF: 108638).

### Instrumented Mouthguards

All players underwent three-dimensional (3D) dental scans and were provided with custom-fit iMGs (Prevent Biometrics, Minneapolis, MN, USA). For technical details surrounding the iMG processes (including fitting, filtering and transformation), see [13, 17] and for validation details, see [18].

### Training and Match Data

Methods of collection of training and match data are detailed elsewhere [13]. Briefly, data were included from the two main rugby training days of the week (i.e. those in which controlled contact, full contact and set piece training were undertaken) during the season and match-days. In this study, the start/finish times and contact type of each drill (non-contact, controlled contact, full contact and set pieces [11]) were coded by two experienced analysts for each training session. For each drill, data were only included if players were wearing their iMG whilst participating. Video analysis data for contact events during match-play were acquired from Opta (StatsPerform, Chicago, IL, USA). Player matches were only included when the instrumented players wore their iMG for at least 90% of their contact events [17]. For simplicity, in addition to the different training contact types, match-play is considered a standalone contact type hereafter.

Daily counts of HAEs and minutes played/trained were produced for each player within each contact type from the coded drills/matches. Days where a high proportion of HAEs included moderate/severe noise, as classified by the iMG manufacturers’ in-house algorithms, were removed from the dataset (>40% noisy from a minimum of five HAEs*, n* = 135). Weekly counts of HAEs and training/playing minutes were calculated as the sum of all drills within each contact type per week. Counts of HAEs were analysed at five magnitudes: *all recorded* HAEs >5 *g* and 400 rads/s^2^; peak linear acceleration (PLA) >10 *g*; PLA >25 *g*; peak angular acceleration (PAA) >1000 rads/s^2^; and PAA >2000 rads/s^2^. The *all recorded* HAEs and >25 *g* and 2000 rads/s^2^ thresholds were modelled in line with previous studies [13, 19]. The >10 *g* and >1000 rads/s^2^ thresholds were added to provide greater granularity within this analysis.

### Statistical Modelling

To estimate the average incidence of HAEs for each position and contact type, generalised linear mixed models were used, assuming a Poisson distribution with a log link. Sex (male or female), position (forward or back), contact type (non-contact training, controlled contact training, set piece training, full-contact training or match-play) and the logarithm of minutes played (to allow for a linear relationship between HAE counts and minutes played, on the logarithmic scale, if the data suggested it was present) were included as fully factorial fixed effects. Random effects were assumed for player ID (to account for repeated measures within players and to estimate player variability for the simulation process) and for week ID (to account for multiple players participating in the same training week or match and to provide a weekly variability estimate for the simulation process). Separate models were run for each of the five HAE magnitudes. The use of the Poisson distribution versus a negative binomial distribution was confirmed using goodness of fit metrics. Likelihood ratio tests confirmed the inclusion of two random effects improved the fit of the model.

### Simulation Process

A simulation approach was used to estimate the overall in-season HAE exposure of men’s and women’s rugby union players. The in-season period was defined as 30 weeks of training and match-play. Individual simulations were conducted for the five HAE magnitudes (*all recorded* HAEs; PLA >10 *g*, PLA >25 *g*; PAA >1000 rads/s^2^; and PAA >2000 rads/s^2^). Using incidence estimates from the generalised linear mixed models described above, samples of weekly HAE counts for 40,000 simulated players were drawn from a Poisson distribution (10,000 for each sex-position combination: men’s forwards, men’s backs, women’s forwards and women’s backs). For brevity, the simulation process is described for a single magnitude for men’s forwards but was repeated for all sex-position combinations across all five magnitudes.

For each contact type, group mean values were estimated using the fixed effects of the models. This resulted in five group mean values, each of which represented the logarithm of the average weekly HAE count for a contact type on the basis of the minutes used in this study. In this study, the upper limits of World Rugby’s 2023 contact load guidelines [11] were used: 40 minutes of controlled contact training, 15 minutes of full-contact training and 30 minutes of set piece training (for forwards only, as backs did not complete any set piece training within this sample). These training times were used as a reference point to contextualise the simulation results. Non-contact training was included as the median minutes trained in the sample, rounded to the nearest 5 minutes, plus 25 minutes as an estimate for the additional minutes of non-contact training a player could experience in training outside of the main sessions included in this study (i.e. those in which controlled contact, full contact and set piece training were not completed). For match-play, the median minutes played within the sample of data per sex-position combination was used for the minutes played. Supplementary Table 1 provides an overview of the fixed effects values used to specify the group mean values across all sex-position combinations, for each simulation.

Next, 10,000 players were simulated using the variability from the player ID random effect. Within the context of the model, a player was represented by an adjustment from the group mean value. Each player adjustment was simulated from a normal distribution of random adjustments, on the logarithmic scale, parameterised by the standard deviation from the player ID random effect (X ~ *N*(0, player ID)). Random adjustments for each player were simulated independently for each HAE magnitude model. Varying the individual player mean values by adjusting from the group mean value in this manner allowed players more or less likely to experience HAEs than the group mean average to be represented within the simulations.

To allow for variability between weeks across the 30-week in-season period, the week ID random effect was used to simulate a random adjustment for each contact type, each week. Two random week adjustments were simulated each week: one for the training contact types, meaning non-contact, controlled contact, full-contact and set piece training all used the same week ID adjustment; and one specifically for match-play. The random adjustments were sampled from a normal distribution (on the logarithmic scale) with mean 0 and standard deviation week ID (X ~ *N*(0, week ID)). Producing the random adjustments in this manner ensured that the training contact types were correlated within weeks (i.e., counts from all contact types were likely to increase or decrease dependent on the week adjustment) and had no relationship with match-play counts.

A player week mean value for each contact type was calculated as the group mean value plus the player adjustment plus the week adjustment. After adjustments were made for player ID and week ID on the logarithmic scale, each player week mean value was exponentiated and a single HAE count was simulated from a Poisson distribution (X ~ *Poisson*(player week mean)) for the contact type. This resulted in 1,500,000 contact type counts for each sex-position combination, simulating a 30-week in-season period of competitive rugby union (150 counts per player, for 10,000 players). The overall in-season HAE exposure for each player was calculated for training as the sum of all HAE counts from training drills (full-contact, controlled contact, set piece and non-contact contact types) for the simulated 30-week in-season period of competitive rugby union. For match-play, it was calculated as the sum of all match-play counts within the simulated 30-week period. Overall rugby minutes were calculated as the sum of all minutes played across the 30-week period, inclusive of training and match-play.

#### Simulating the Impact of Changing Weekly Contact Duration Beyond World Rugby Contact Guidelines

**Fig. 1:**
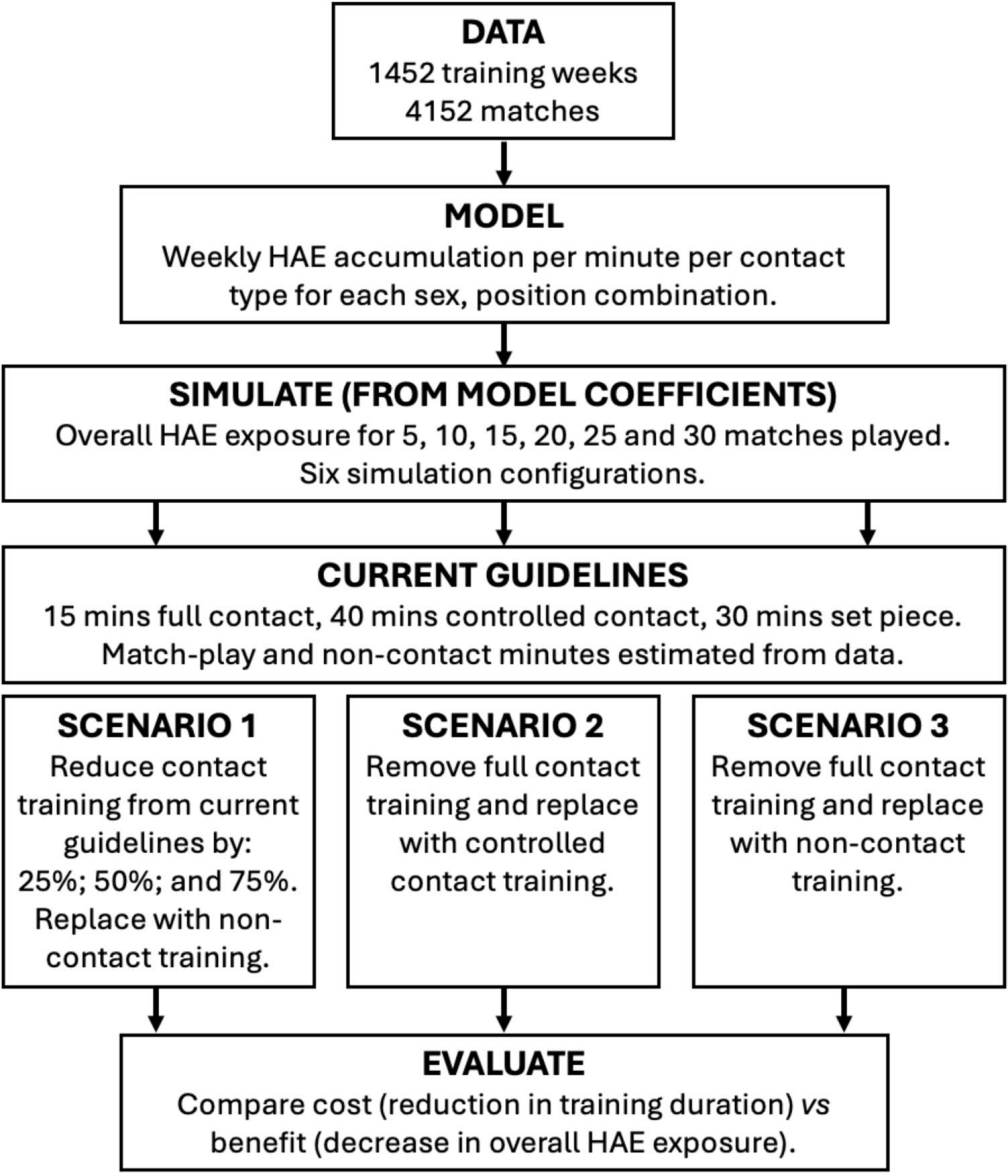
Schematic of the study design

To evaluate the impact of changing weekly contact training duration beyond World Rugby contact load guidelines on overall in-season HAE exposure, six simulation configurations were tested across three scenarios. Current World Rugby contact load guidelines were used to provide a reference duration for weekly contact duration (i.e. 15 minutes full-contact training, 40 minutes controlled contact training and 30 minutes set piece training, with non-contact training minutes estimated from the data). In scenario 1, three contact training duration reductions were considered: reducing weekly contact training by 25%, 50% and 75% beyond current guidelines. In each of these configurations, contact training minutes were reduced for each of full-contact, controlled contact and set piece training and replaced with non-contact training as teams would likely still train for the same duration if guidelines were altered [9]. In scenarios 2 and 3, the substitution of full-contact training with other training types was assessed. In scenario 2, the 15 minutes of full-contact training were assigned to controlled contact training; in scenario 3, the full-contact minutes were substituted for non-contact training. Figure 1 shows the analysis and simulation procedure for this study. Supplementary Table 1 provides the minutes used for each of the simulations.

To get a broad understanding of the impact of changing training across the population of players, separate simulations were run for each HAE magnitude (*all recorded* HAEs; PLA >10 *g*, PLA >25 *g*; PAA >1000 rads/s^2^; and PAA >2000 rads/s^2^) and across six seasonal match exposures. The seasonal match exposures considered were 5, 10, 15, 20, 25 and 30 matches played, at the median minutes played per positional group per match. For each seasonal match exposure, it was assumed that the player completed 30 full weeks of in-season training. For all seasonal match exposures where players did not participate in 30 matches, the matches a player missed were randomly selected across the simulated season. The HAE counts and minutes played from these randomly selected matches were set to 0.

### Presentation of Results

The simulations in this study provide expected counts of HAEs, rounded to whole numbers because it is not possible to experience partial HAEs. The results focus on median values and differences for overall HAE exposure. Where average differences are provided, these are the rounded mean of median absolute or percentage reductions in HAEs across the six seasonal match exposures. Reference intervals for all simulations, which provide a range of overall HAE exposures a player could be expected to experience during a 30-week period of in-season competitive rugby union, are provided in the supplementary material (Supplementary Tables 2–11). Supplementary Figs. 1–5 plot the models used to simulate the results of this study. All models and simulations were conducted in R using the *glmmTMB* package [20] for the generalised linear mixed models and custom algorithms for the simulations.

## Results

**Fig. 2:**
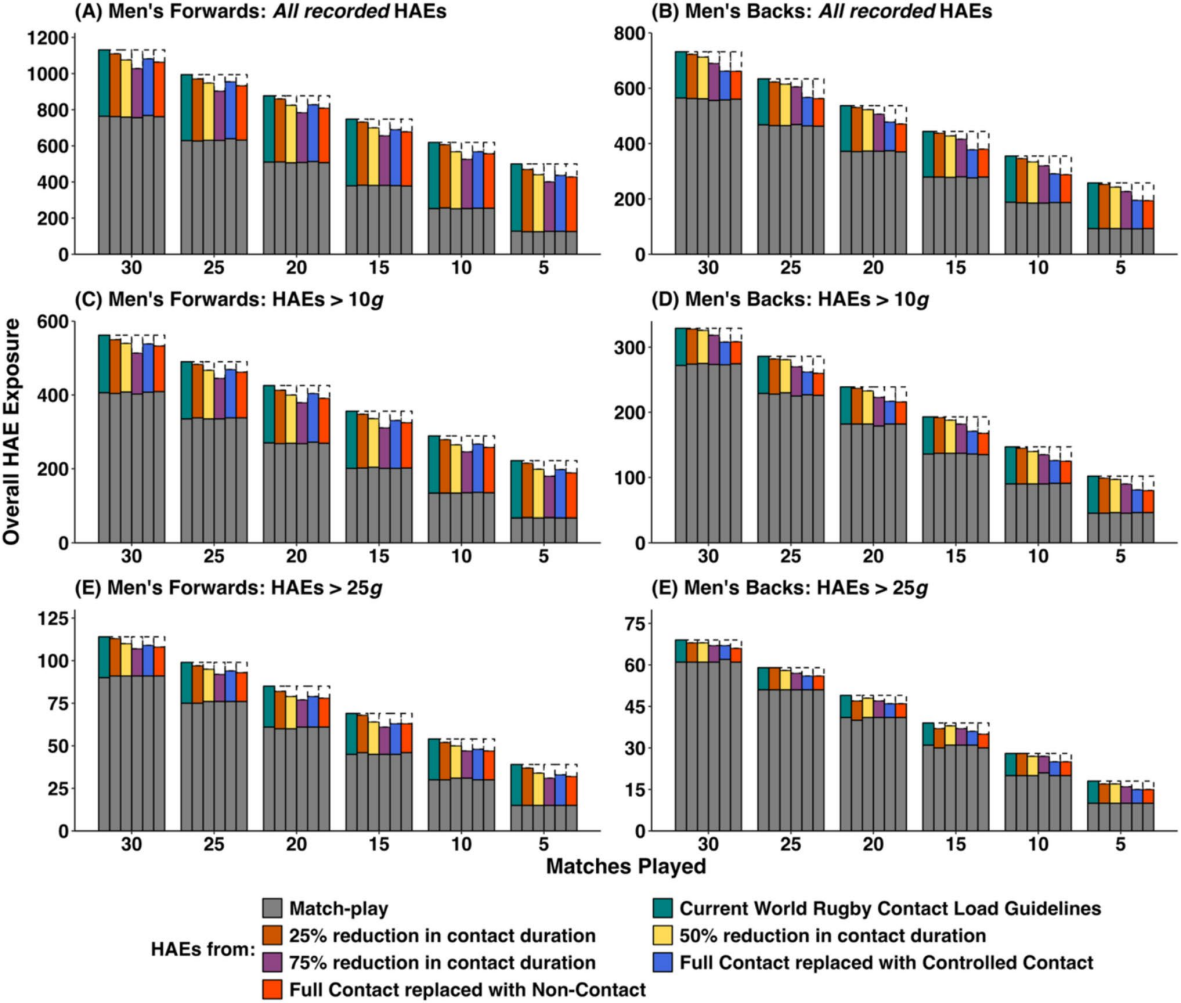
Simulated effect of reducing contact training beyond World Rugby contact load guidelines on overall in-season HAE exposure in men’s rugby union (PLA only). Data are median values, dashed boxes represent reductions in HAEs compared with current guidelines. *Note*: *y*-axes scales differ, thus visual comparison between forwards and backs is not advised

**Fig. 3:**
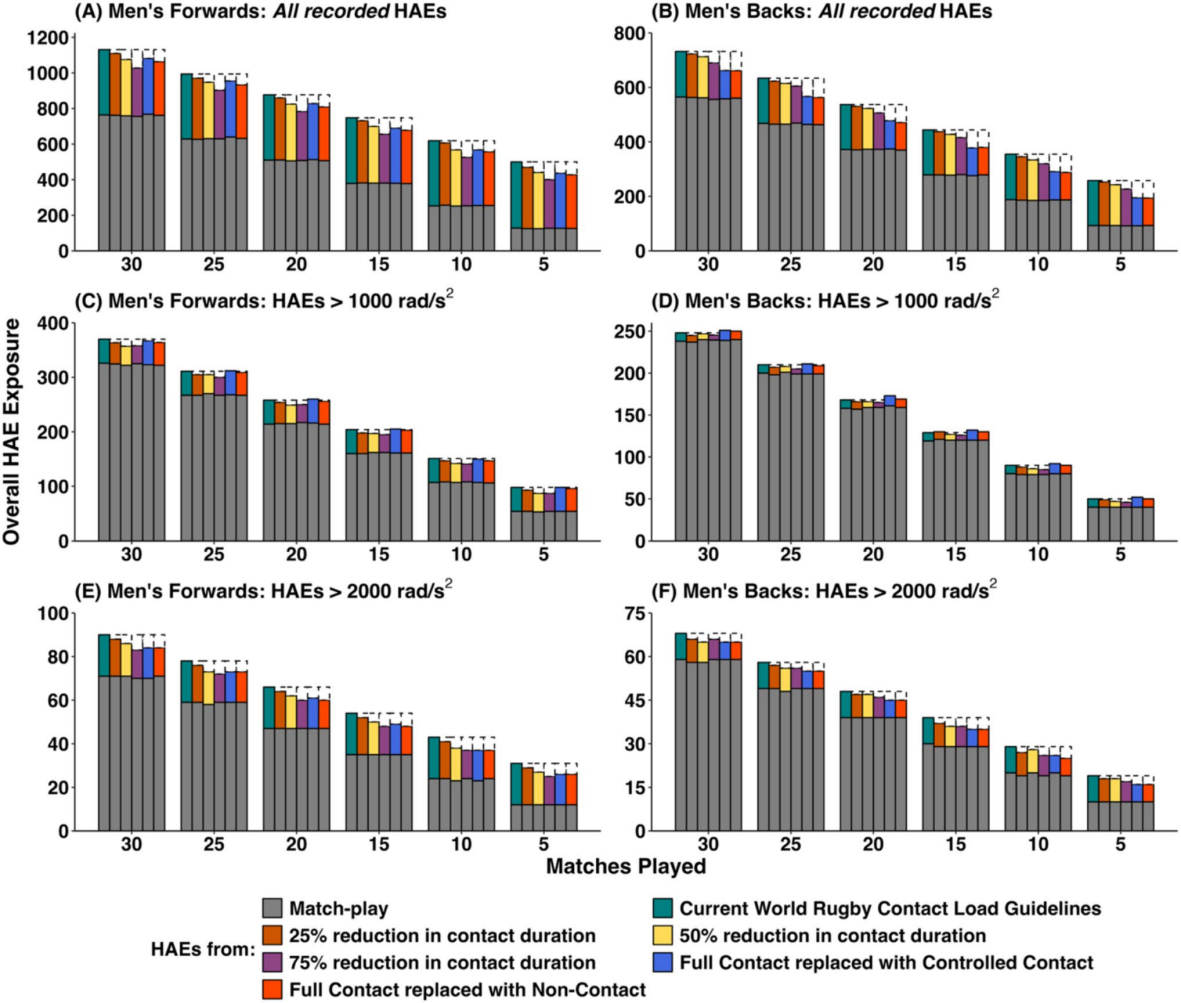
Simulated effect of reducing contact training beyond World Rugby contact load guidelines on overall in-season HAE exposure in men’s rugby union (PAA only). Data are median values, dashed boxes represent reductions in HAEs compared with current guidelines. *Note*: *y*-axes scales differ, thus visual comparison between forwards and backs is not advised

**Fig. 4:**
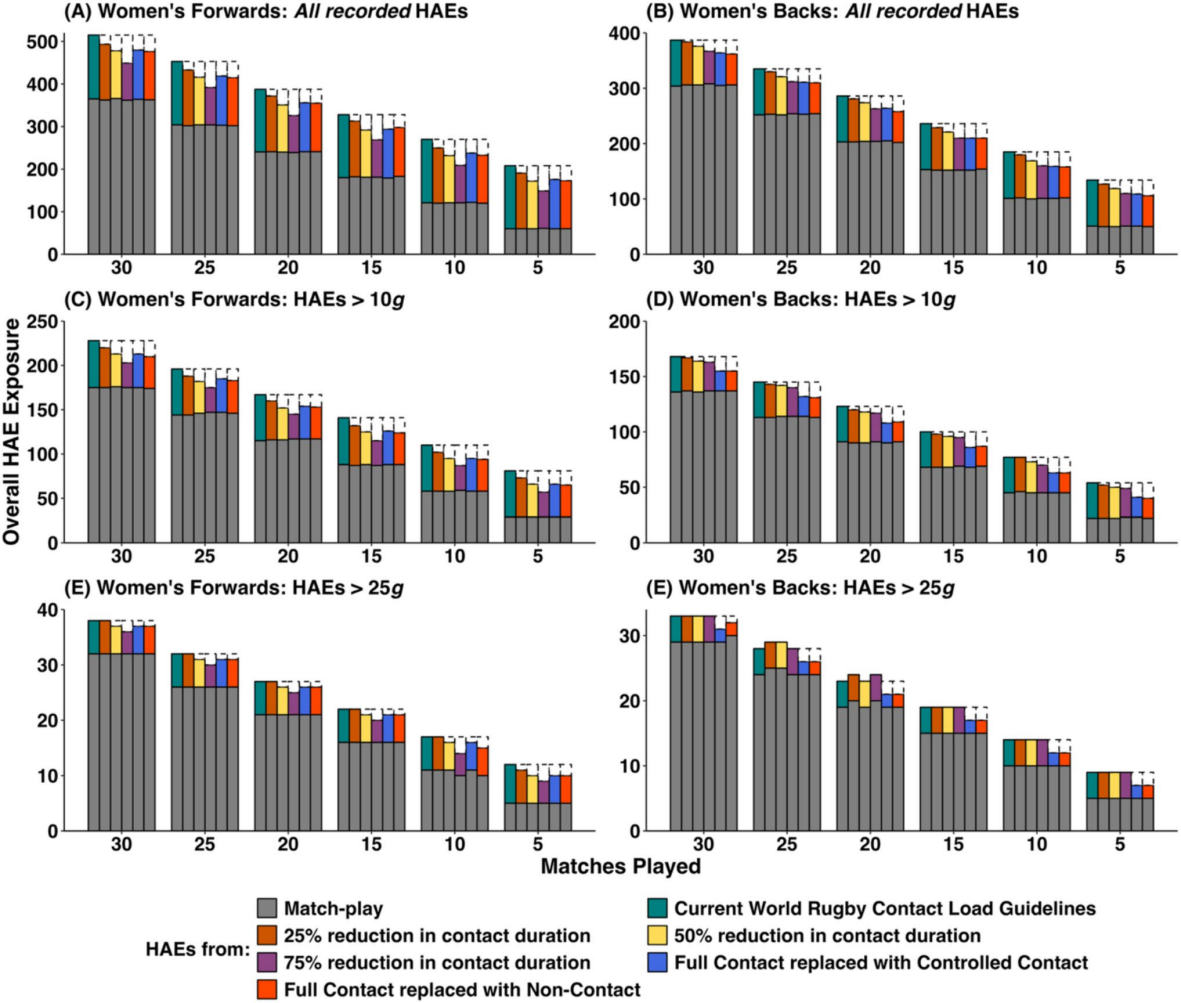
Simulated effect of reducing contact training beyond World Rugby contact load guidelines on overall in-season HAE exposure in women’s rugby union (PLA only). Data are median values, dashed boxes represent reductions in HAEs compared with current guidelines. *Note*: *y*-axes scales differ, thus visual comparison between forwards and backs is not advised

**Fig. 5:**
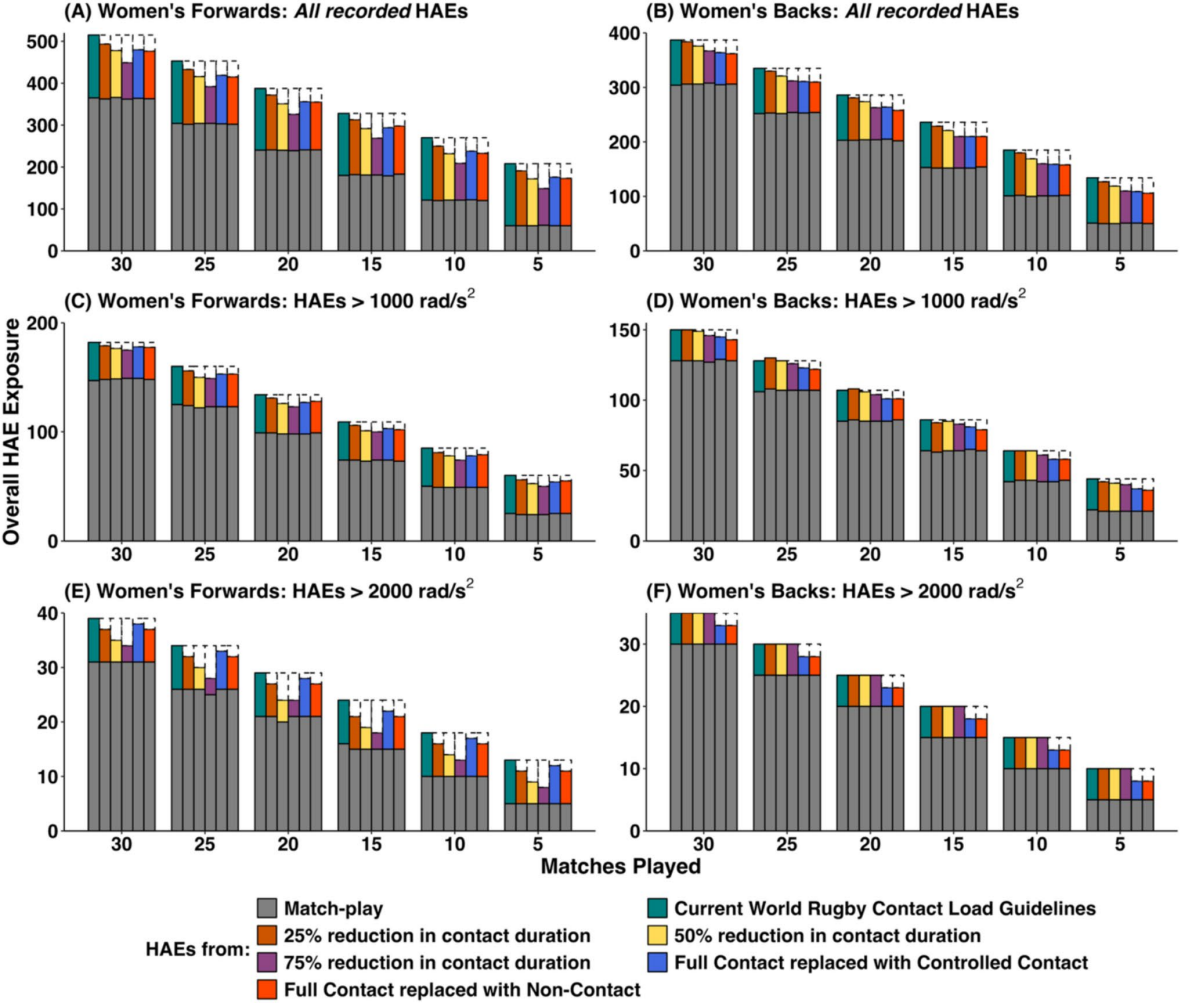
Simulated effect of reducing contact training beyond World Rugby contact load guidelines on overall in-season HAE exposure in women’s rugby union (PAA only). Data are median values, dashed boxes represent reductions in HAEs compared with current guidelines. *Note*: *y*-axes scales differ, thus visual comparison between forwards and backs is not advised

Figure 2 (PLA – men), Fig. 3 (PAA – men), Fig. 4 (PLA – women) and Fig. 5 (PAA – women) depict the effect of changing weekly contact training relative to the World Rugby contact load guidelines in rugby union across a range of seasonal match exposures (i.e. 5, 10, 15, 20, 25 or 30 matches played) and reductions (i.e. 25%, 50% and 75% reductions in contact training durations, and replacing full-contact training with controlled or non-contact training). Table 1 provides mean HAE decreases across the simulations in absolute and percentage terms. Overall HAE exposure was decreased across all simulation configurations by a maximum of 3.2 HAEs per week (*all recorded*), 1.5 HAEs per week (>10 *g*), 0.3 HAEs per week (>25 *g*), 0.3 HAEs per week (>1000 rad/s^2^) and 0.2 HAEs per week (>2000 rad/s^2^). Players participating in fewer matches experienced a greater proportional decrease in HAEs. For example, at a 75% reduction in contact training, the average percentage decrease in *all recorded* HAEs for men’s forwards was 12.7%, but the decrease was 9.1% for players participating in 30 matches compared with 19.8% for players participating in 5 matches.

**Table 1:** Mean raw and percentage decrease in contact minutes and HAEs compared with current World Rugby contact load guidelines. Percentages are proportions of overall rugby minutes and HAEs (i.e. inclusive of both training and match-play)

	Contact minutes		All recorded HAEs		HAEs > 10 *g*		HAEs > 25 *g*		HAEs > 1000 rad/s^2^		HAEs > 2000 rad/s^2^	
	Raw	%	Raw	%	Raw	%	Raw	%	Raw	%	Raw	%
**Men’s forwards**												
25% reduction	637.5	13.7	20	2.7	9	2.6	2	2.8	5	2.7	2	3.8
50% reduction	1275	27.3	52	7.0	23	6.5	5	7.0	9	4.9	4	8.1
75% reduction	1912.5	41.0	95	12.7	45	12.5	8	11.3	10	5.4	6	11.5
FC replaced with CC	0	0	52	7.1	23	6.5	6	8.6	0	0.0	5	10.0
FC replaced with NC	450	9.6	67	8.9	31	8.9	7	9.9	3	1.4	6	10.6
**Men’s backs**												
25% reduction	412.5	8.8	8	1.7	2	1.2	1	2.7	2	1.2	2	4.0
50% reduction	825	17.7	17	3.9	5	2.9	1	2.8	2	2.4	2	4.4
75% reduction	1237.5	26.5	33	7.4	13	6.9	2	5.0	4	3.5	2	6.5
FC replaced with CC	0	0	65	14.7	22	11.7	3	8.2	− 3	− 2.2	3	8.7
FC replaced with NC	450	9.6	67	15.2	23	12.4	3	8.9	− 1	− 0.3	3	9.3
**Women’s forwards**												
25% reduction	637.5	13.7	18	5.5	8	5.9	0	1.4	4	3.4	2	9.5
50% reduction	1275	27.3	37	11.2	15	11.0	1	6.1	8	7.2	4	18.8
75% reduction	1912.5	41.0	61	18.5	24	17.3	2	11.8	10	9.5	5	23.2
FC replaced with CC	0	0	14	10.0	14	10.5	1	6.1	6	5.9	1	5.1
FC replaced with NC	450	9.6	16	10.7	16	11.5	1	7.1	6	5.5	2	9.5
**Women’s backs**												
25% reduction	412.5	8.8	5	2.5	2	1.7	0	1.3	0	0.7	0	0.0
50% reduction	825	17.7	14	6.2	4	4.2	0	0.6	1	1.6	0	0.0
75% reduction	1237.5	26.5	14	10.4	6	5.8	0	0.7	3	4.0	0	0.0
FC replaced with CC	0	0	24	10.8	14	14.2	2	11.5	6	8.4	2	10.6
FC replaced with NC	450	9.6	27	11.7	14	14.3	2	11.0	7	7.3	2	10.6

*N.B.* reduction (25%, 50%, 75%) in contact durations from current guidelines and replaced with non-contact training; *FC* full contact, *CC* controlled contact; *NC* non-contact training. Negative signs removed from decreases for readability

### Scenario 1: Reducing All Contact Load Durations Beyond Current World Rugby Guidelines and Replacing with Non-contact Training

There was a dose-dependent decrease in overall HAE exposure when reducing contact load duration beyond current World Rugby guidelines. However, the absolute decrease in HAEs was small (ranging from 2 HAEs >25 *g* per season for a 25% reduction in contact training to 95 *all recorded* HAEs per season at a 75% reduction in contact training for men’s forwards). This decrease was disproportionately lower than the reductions in contact training durations required to achieve it. For example, in men’s forwards a 25% reduction in contact training duration from current World Rugby guidelines (equivalent to 10.63 hours or 13.7% of overall rugby minutes decreased HAE exposure for *all recorded* HAEs by 2.7% (20), >10 *g* HAEs by 2.6% (9), >25 *g* HAEs by 2.8% (2), >1000 rad/s^2^ HAEs by 2.7% (5) and >2000 rad/s^2^ HAEs by 3.8% (2). A 50% reduction in contact training duration, equivalent to 21.25 hours or 27.3% of overall rugby minutes, decreased HAE exposure for *all recorded* HAEs by 7.0% (52 HAEs), >10 *g* HAEs by 6.5% (23 HAEs), >25 *g* HAEs by 7.0% (5 HAEs), >1000 rad/s^2^ HAEs by 4.9% (9 HAEs) and >2000 rad/s^2^ HAEs by 8.1% (4 HAEs). A 75% reduction in contact training duration (equivalent to 31.88 hours or 41.0% of overall rugby minutes) decreased HAE exposure for *all recorded* HAEs by 12.7% (95 HAEs), >10 *g* HAEs by 12.5% (45 HAEs), >25 *g* HAEs by 11.3% (8 HAEs), >1000 rad/s^2^ HAEs by 5.4% (10 HAEs) and >2000 rad/s^2^ HAEs by 11.5% (6 HAEs).

### Scenario 2: Replacing Full Contact Training with Controlled Contact Training

In scenario 2, there was no reduction in total contact training duration compared with current guidelines, as full-contact training was replaced with the lower intensity controlled contact training but decreases in HAEs were still observed. In women’s forwards, HAE exposure was decreased for *all recorded* HAEs by 10.0% (14 HAEs), >10 *g* HAEs by 10.5% (14 HAEs), >25 *g* HAEs by 6.1% (1 HAE), >1000 rad/s^2^ HAEs by 5.9% (6 HAEs) and >2000 rad/s^2^ HAEs by 5.1% (1 HAE). In women’s backs, HAE exposure was decreased for *all recorded* HAEs by 10.8% (24 HAEs), >10 *g* HAEs by 14.2% (14 HAEs), >25 *g* HAEs by 11.5% (2 HAEs), >1000 rad/s^2^ HAEs by 8.4% (6 HAEs) and >2000 rad/s^2^ HAEs by 10.6% (2 HAEs).

### Scenario 3: Replacing Full-Contact Training with Non-Contact Training

In scenario 3, contact training durations were reduced by 7.5 hours (9.6% of overall rugby minutes). The decreases in HAE exposure were approximately proportional to the contact training duration reduction, with the exception of HAEs > 1000 rad/s^2^ in men’s rugby. For example, in men’s forwards, HAE exposure was decreased for *all recorded* HAEs by 8.9% (67 HAEs), >10 *g* HAEs by 8.9% (31 HAEs), >25 *g* HAEs by 9.9% (7 HAEs), >1000 rad/s^2^ HAEs by 1.4% (3 HAEs) and >2000 rad/s^2^ HAEs by 10.6% (6 HAEs). In women’s forwards, HAE exposure was decreased for *all recorded* HAEs by 10.7% (16 HAEs), >10 *g* HAEs by 11.5% (16 HAEs), >25 *g* HAEs by 7.1% (1 HAE), >1000 rad/s^2^ HAEs by 5.5% (6 HAEs) and >2000 rad/s^2^ HAEs by 9.5% (2 HAEs).

## Discussion

The aim of this study was to use simulation techniques to evaluate the effect of changing weekly contact durations relative to current World Rugby contact load guidelines [11] on overall in-season HAE exposure, across a range of training reductions (25%, 50% and 75% decreases in training duration compared with current guidelines; and replacing-full contact training with controlled contact or non-contact training) and match exposures (5, 10, 15, 20, 25 and 30 matches played). The results showed that all simulation configurations reduced overall HAE exposure in absolute terms by a maximum of 3.2 HAEs per week (95 *all recorded* HAEs per season or 12.7% of HAEs in men’s forwards at a 75% reduction in contact training duration), but this decrease was disproportionately greater in players who played fewer matches (e.g. 9.1% decrease for players playing 30 matches versus 19.8% for players playing 5 matches). In scenario 1 (reducing contact training duration compared with current guidelines by 25%, 50% and 75%), there was a dose-dependent decrease in HAE exposure but the decrease in HAEs was disproportionately lower than the reduction in contact training duration. For example, in men’s forwards, a 50% reduction in contact training decreased the duration of overall rugby minutes by 27.3% but only decreased HAEs by ~7%. Scenario 2 (replacing full-contact training with controlled contact training) was at least as effective at decreasing HAEs as reducing all contact training by 50% and did not require contact training duration to be reduced. Scenario 3 (replacing full contact training with non-contact training) was slightly more effective than scenario 2 at decreasing HAEs but required contact training duration to be reduced by 9.6% of overall rugby minutes compared with current guidelines. This study provides important context for consideration in the management of HAEs through potential contact training modifications.

### Scenario 1: Reducing All Contact Load Durations Beyond Current World Rugby Guidelines and Replacing with Non-contact Training

The results of this study show that, on average across all sex and position combinations, overall in-season exposure to HAEs >10 *g* would decrease by 2.9% (5 HAEs), 6.2% (12 HAEs) and 10.6% (22 HAEs) if contact training duration was reduced by 25%, 50% or 75% compared with current guidelines. Sports are aiming to reduce HAEs, thus any decrease could be considered positive. However, the magnitude of decrease provided by these reductions in contact training time was limited. These findings contrast with previous research in American football [9], where the removal of one contact session per week was expected to reduce overall seasonal HAE exposure by 138 or 18% (no duration was provided for the contact session). The discrepancy between these studies can be explained by the differences in the contributions made by matches and training to total weekly HAEs. In professional rugby union, at current World Rugby guidelines, a very small proportion of HAEs occur during training (for example, men’s forwards experienced 9 HAEs per training week compared with 25 HAEs per match using an 8 *g* trigger threshold [13]). By contrast, high school American football players experience considerably more HAEs in training despite using a higher 14.4*g* trigger threshold, with as many as 16.5 HAEs in a *single* contact session previously reported [9]. Given the low contribution of training to HAE exposure in professional rugby union [13, 14], it is unsurprising that our simulations show only small reductions in overall HAE exposure. To achieve the same decrease in HAEs in professional rugby union as were achieved in high school American football, all training would have to be removed completely.

The decrease in HAEs simulated in scenario 1 was also disproportionately smaller than the training reduction. For example, reducing contact training by 50% required a 27.3% reduction in overall rugby minutes for ~ 7% decrease in HAEs in men’s forwards. This is also a consequence of higher HAE incidences being observed in matches than training. Indeed, in scenario 1 simulations, a reduction of 5 matches (or 4.25 hours of rugby) predicted a reduction of ~ 65 HAEs >10 *g* for men’s forwards, which is almost 50% more than the decrease obtained from reducing contact training duration by 75% or 31.88 hours across the season. Taken together, the limited benefits to HAEs and disproportionate cost of reducing contact training time highlight scenario 1 as a relatively inefficient method of reducing HAEs in professional rugby union.

### Scenarios 2 and 3: Replacing Full-contact Training with Other Training Types

In scenarios 2 and 3, an alternative approach of replacing full contact training with controlled contact training or non-contact training was evaluated. Both simulations were at least as effective as reducing all contact training by 50% at decreasing HAE exposure and required significantly lower reductions in contact training time. However, both removed all full-contact training, which would mean that players only experience full-contact rugby during match-play. Previous research has shown that players who experience injuries during contact events demonstrate lower tackling and ball-carrying technical proficiency [21], thus it is essential that these contact skills are developed and maintained across a range of training activities from controlled contact through to full-contact training [22]. Removing the opportunity for players to maintain their technical proficiency within contact when they are not playing matches could therefore be seen as an imperfect strategy if applied at a population level. However, for players who regularly experience full-contact rugby whilst playing matches and who are experiencing high numbers of HAEs, replacing full-contact training with controlled contact training for a prescribed time period could be useful in allowing time for contact technique to be improved at lower intensities whilst also reducing HAE exposure from training.

### Real-World versus Simulated Outcomes

It is important to note that a multitude of factors can influence the success of policy changes compared with simulated outcomes. From a practical perspective, understanding stakeholder compliance is particularly important given that studies have shown that 30–50% of coaches utilise full contact training at durations greater than the upper limit of the current World Rugby contact load guidelines [11, 23]. Therefore, it is also worth noting that if changes to the guidelines were enforced as policy, significant resources would be required to monitor compliance. In the context of the decreases in HAEs shown in this study, the cost of the resources required to manage a change may outweigh any potential benefits. Future studies can begin to model barriers to implementation quantitatively through sensitivity analyses of partial compliance or delayed adoption, or a systems-based approach to analysis [24], enabling more accurate decisions to be made regarding the potential efficacy of policy decisions.

### Limitations and Future Directions

The extrapolation of modelled estimates through simulation as shown in this study provides an exciting avenue through which policymakers and researchers can begin to evaluate potential policy changes longitudinally, whilst only collecting samples of data. However, the accuracy of these simulations is directly related to how well the data collected represent the underpinning data generating process [25]. In this study, three key factors may have influenced our results.

The first is the representativeness of the sample on which the simulations are based. The players involved in this study could be considered early adopters to iMGs as they participated in World Rugby’s initial rollout of the technology. As such, they are not a random sample of players and their individual characteristics may not be representative of the whole rugby player population. The second factor is that the models did not evaluate whether a relationship was present between training and match HAEs within a given week. There were not enough data to consider this within our study, but it is possible that on weeks where players are expected to play less, they may train more (e.g. above the contact load guidelines used in this study). This could influence the number of HAEs they experience. Researchers may wish to model this directly in future simulations. Finally, all players were considered to play and train for the same duration, which is not an accurate representation of players’ annual exposure. Future studies can build upon the work in this study to improve the complexity and accuracy of simulations (e.g. simulating the number of minutes a player plays rather than considering the median playing time only). Furthermore, understanding and incorporating the intricacies of specific drills (e.g. collision density [11]), rather than solely considering the overarching contact level, could improve the accuracy of simulations.

Away from training and match-play restrictions, other approaches to reducing HAEs should be evaluated. Examples of these include the use of protective equipment to attenuate the magnitude of HAEs, improvements in technique within contact events, either through coaching or law changes, and the identification and management of players who have higher HAE exposure.

## Conclusions

This is the first study to use simulation techniques to extrapolate model results from a sample of data to evaluate the impact of a change in weekly contact training durations relative to World Rugby contact load guidelines on HAE accumulation. The results show that reducing contact training durations would decrease HAEs but these changes would be small (maximum 3.2 HAE decrease per week; 95 *all recorded* HAEs per season or 12.7% of HAEs in men’s forwards at a 75% reduction in contact training duration), as a result of the current relatively small contribution of training to a player’s weekly HAE total. Reducing contact training would also involve tradeoffs, which policymakers must consider before implementation. The balance between the potential costs and benefits suggests that reducing World Rugby contact load guidelines at a population level would be an inefficient method of decreasing HAEs. However, the removal of full contact training as a method of managing individuals with high match and HAE exposure may be useful.

## Supplementary Information

Below is the link to the electronic supplementary material.

Supplementary file1 (PDF 1960 KB)

## Declarations

### Availability of Data and Material

Data are available upon reasonable request from the corresponding author.

### Code Availability

Code is available upon reasonable request from the corresponding author.
