# Supplementary material for: The Effect of Changing Weekly Contact Training Duration Beyond Current Guidelines on Head Acceleration Events in Rugby Union
Source: Sports Med. 2025 Nov 27;56(4):1043–55. doi: 10.1007/s40279-025-02359-3 (PMC13124761; doi:10.1007/s40279-025-02359-3)
Supplement: Supplementary file 1 — Supplementary file1 (PDF 1960 KB) [file 40279_2025_2359_MOESM1_ESM.pdf]

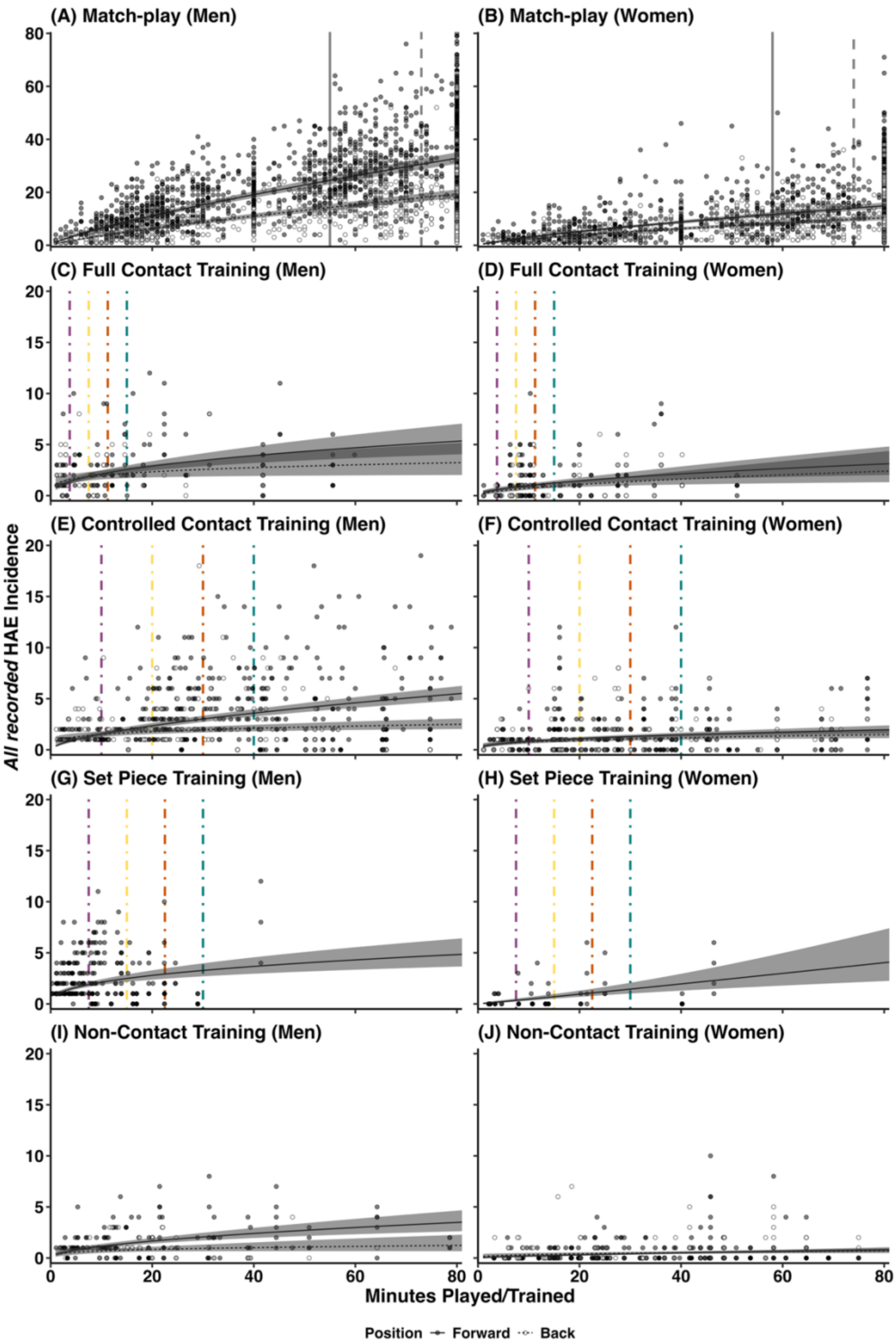

Supplementary Figure 1: *All recorded* HAE model from which simulations were run. Lines represent model estimated mean HAE incidence per minute, shaded areas represent 95% confidence intervals. Dots represent individual training / match weeks. Vertical dashed lines represent times used for simulations: World Rugby contact load guidance (teal), 25% reduction (red), 50% reduction (yellow), 75% reduction (purple).

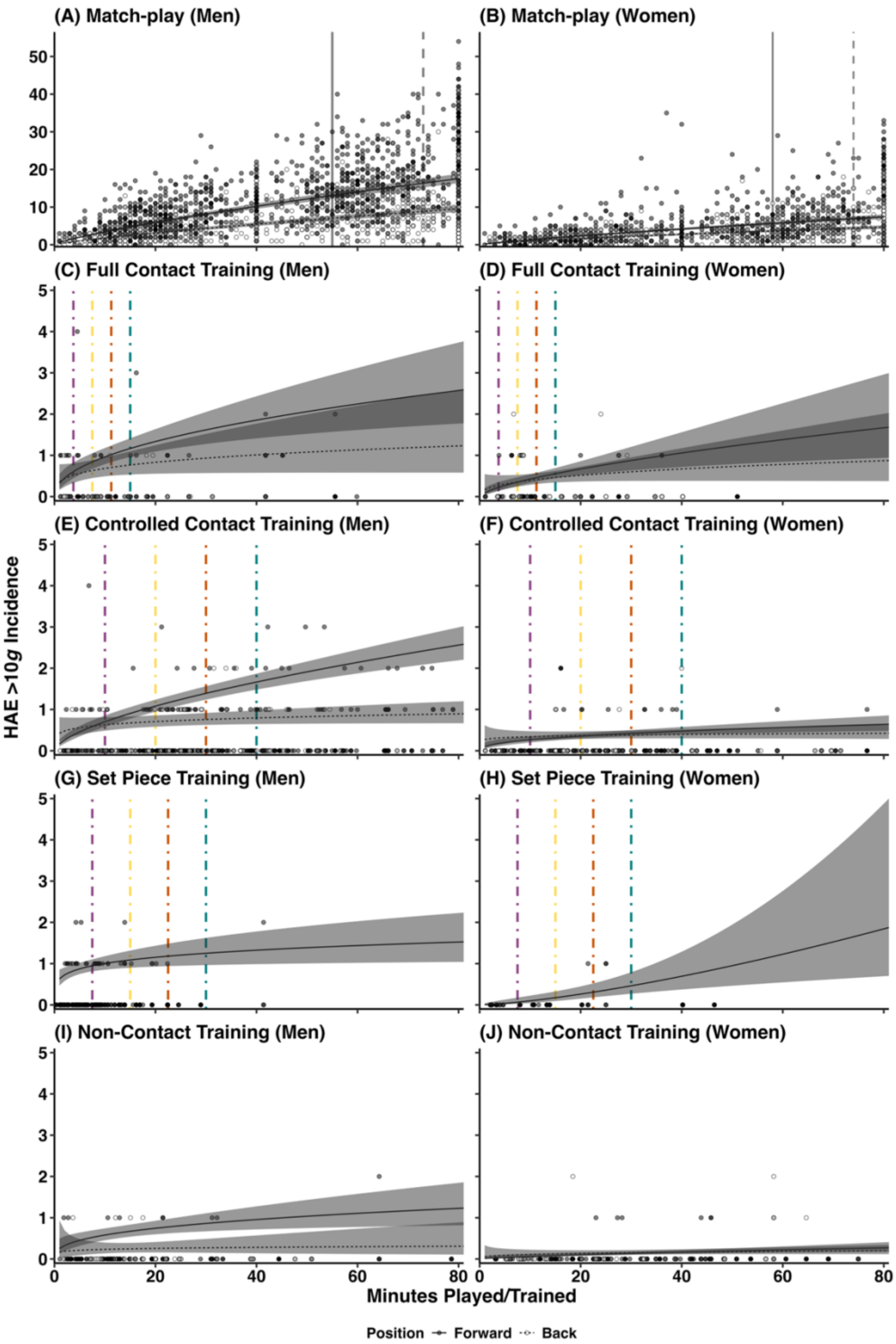

Supplementary Figure 2: HAE >10*g* model from which simulations were run. Lines represent model estimated mean HAE incidence per minute, shaded areas represent 95% confidence intervals. Dots represent individual training / match weeks. Vertical dashed lines represent times used for simulations: World Rugby contact load guidance (teal), 25% reduction (red), 50% reduction (yellow), 75% reduction (purple).

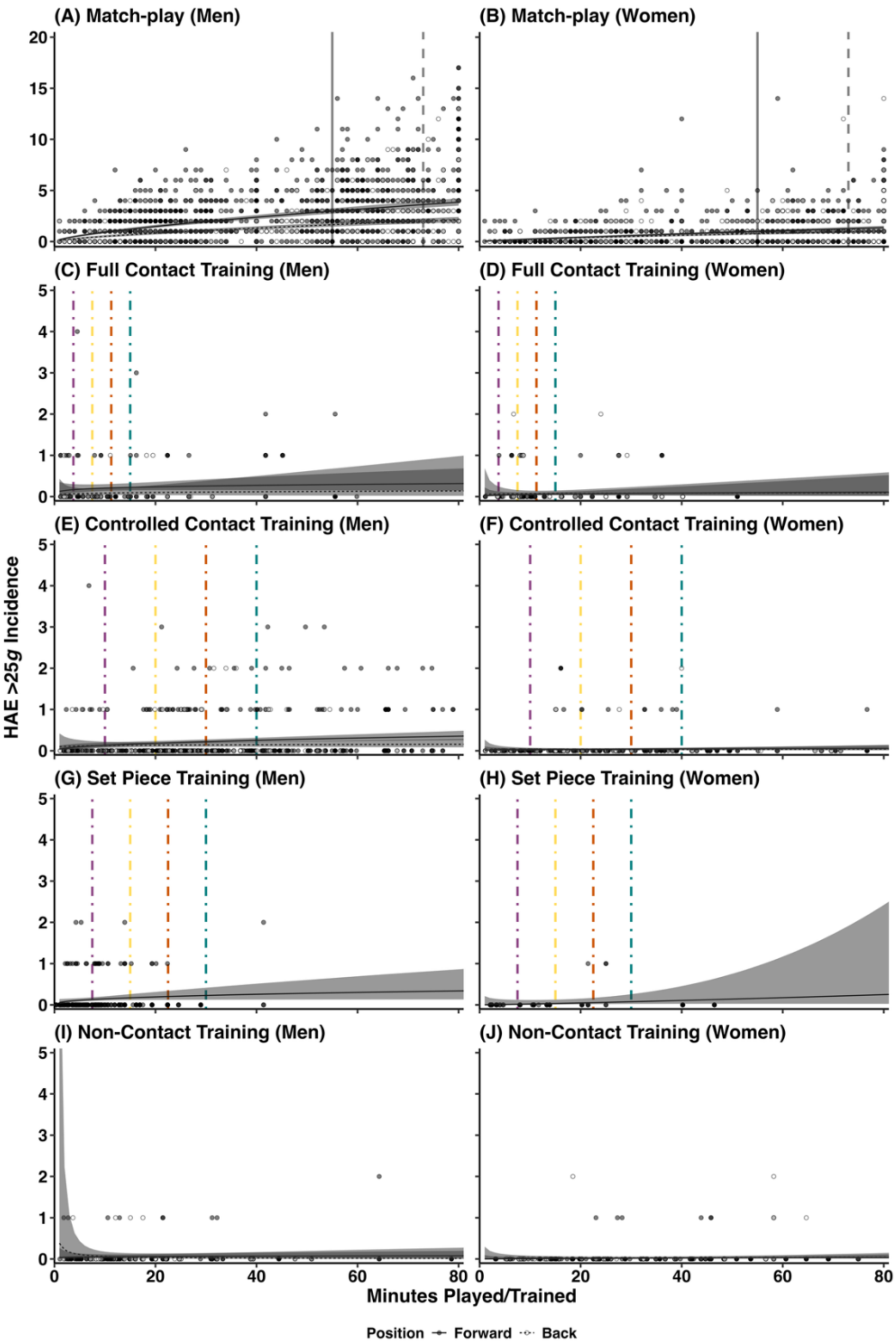

Supplementary Figure 3: HAE >25*g* model from which simulations were run. Lines represent model estimated mean HAE incidence per minute, shaded areas represent 95% confidence intervals. Dots represent individual training / match weeks. Vertical dashed lines represent times used for simulations: World Rugby contact load guidance (teal), 25% reduction (red), 50% reduction (yellow), 75% reduction (purple).

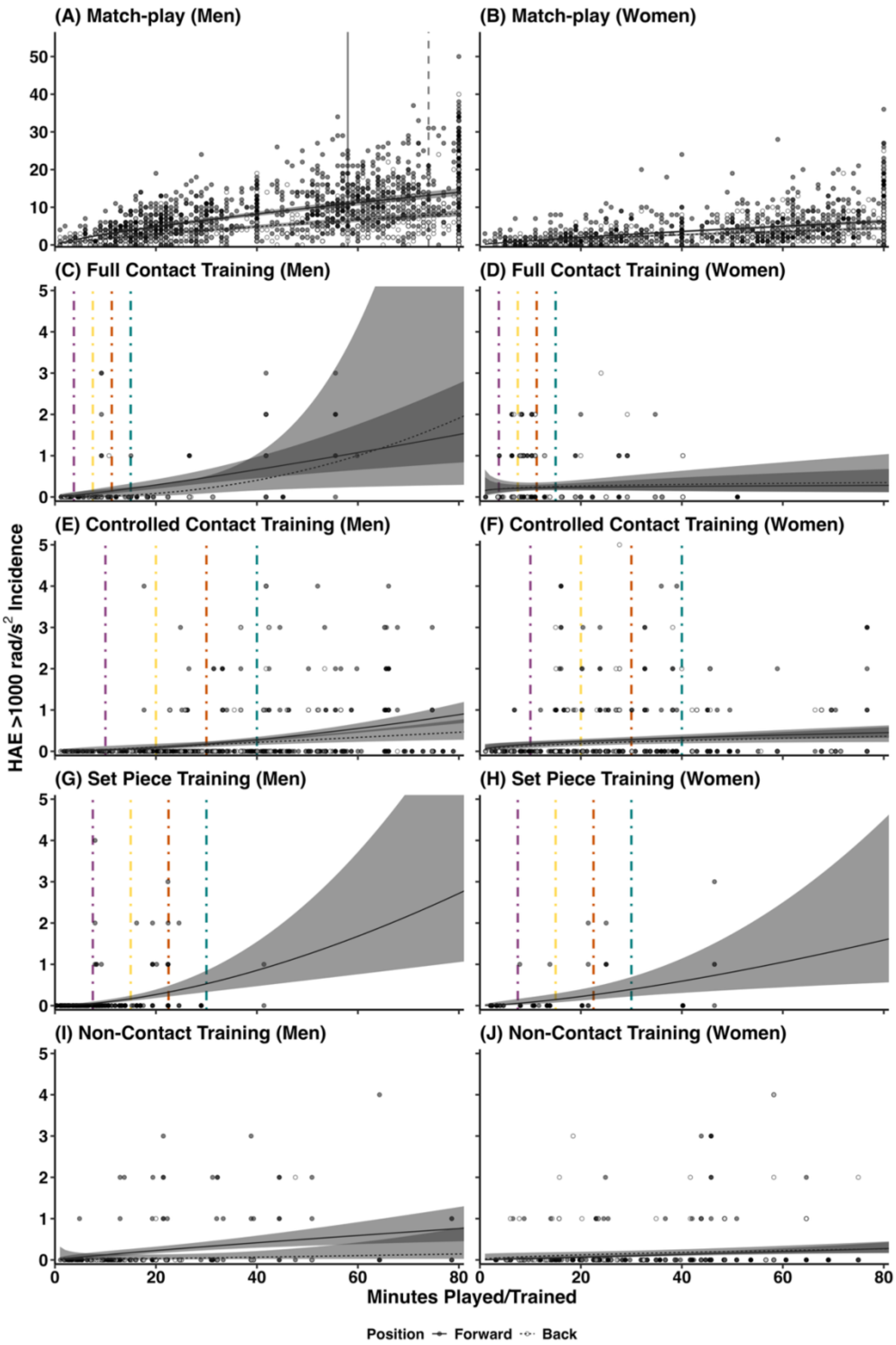

Supplementary Figure 4: HAE >1000 rad/s^2 model from which simulations were run. Lines represent model estimated mean HAE incidence per minute, shaded areas represent 95% confidence intervals. Dots represent individual training / match weeks. Vertical dashed lines represent times used for simulations: World Rugby contact load guidance (teal), 25% reduction (red), 50% reduction (yellow), 75% reduction (purple).

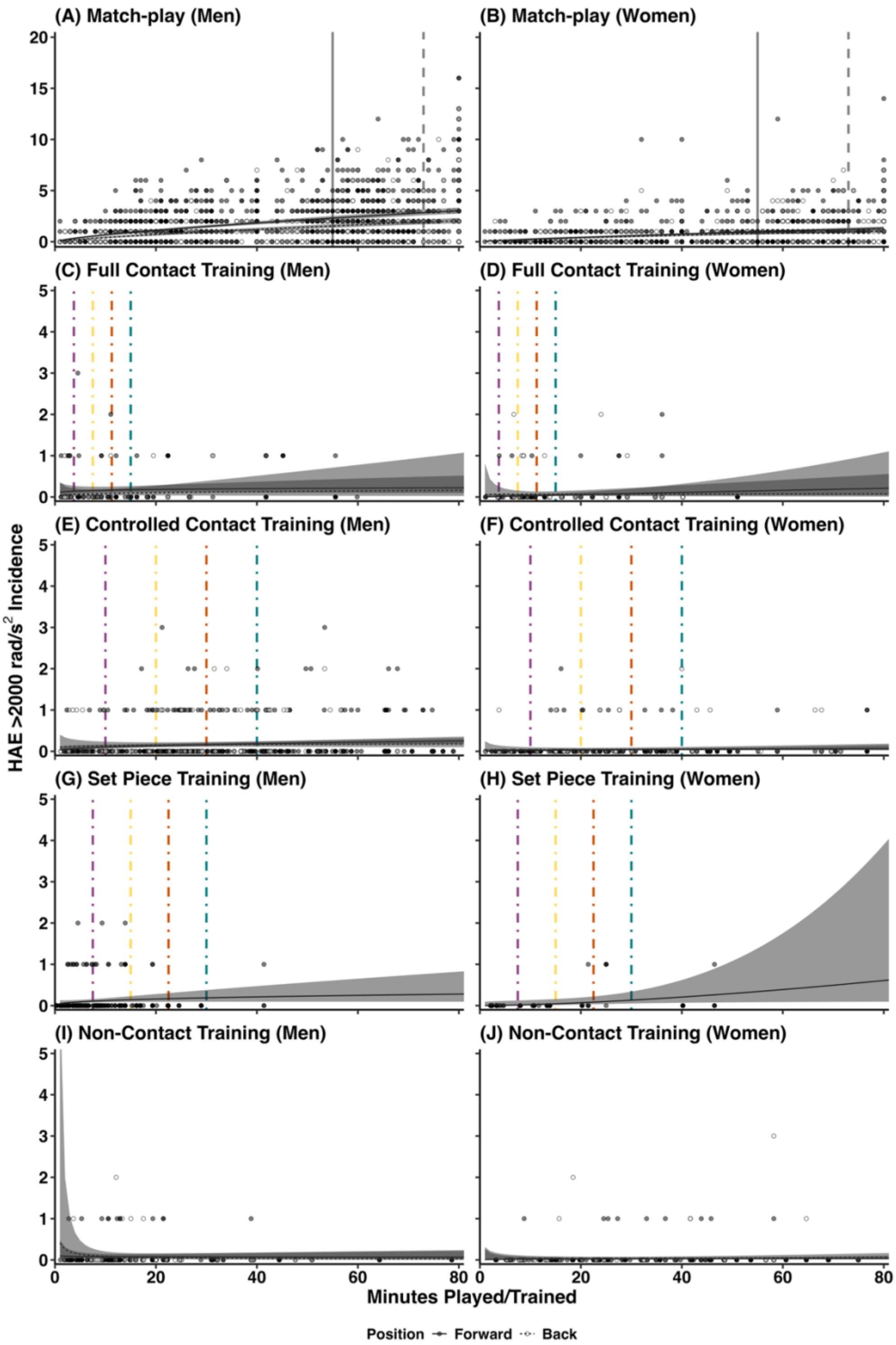

Supplementary Figure 5: HAE >2000 rad/s^2 model from which simulations were run. Lines represent model estimated mean HAE incidence per minute, shaded areas represent 95% confidence intervals. Dots represent individual training / match weeks. Vertical dashed lines represent times used for simulations: World Rugby contact load guidance (teal), 25% reduction (red), 50% reduction (yellow), 75% reduction (purple).

Supplementary Table 1: Fixed effects for group mean lambda values for six simulation configurations. Values represent trainin
minutes. All configurations assume the same total training duration.

Sex	Position	Contact type	$WRCLG_{current}$	$WRCLG_{-25\%}$	$WRCLG_{-50\%}$	$WRCLG_{-75\%}$	$WRCLG_{FC\text{-}CC}$	$WRCLG_{FC\text{-}NC}$
Men	Forward	Non-Contact	40	61.25	82.5	103.75	40	55
Men	Forward	Controlled Contact	40	30	20	10	55	40
Men	Forward	Full Contact	15	11.25	7.5	3.75	0	0
Men	Forward	Set Piece	30	22.5	15	7.5	30	30
Men	Forward	Match-play	55	55	55	55	55	55
Men	Back	Non-Contact	45	58.75	72.5	86.25	45	60
Men	Back	Controlled Contact	40	30	20	10	55	40
Men	Back	Full Contact	15	11.25	7.5	3.75	0	0
Men	Back	Set Piece	0	0	0	0	0	0
Men	Back	Match-play	73	73	73	73	73	73
Women	Forward	Non-Contact	55	76.25	97.5	118.75	55	70
Women	Forward	Controlled Contact	40	30	20	10	55	40
Women	Forward	Full Contact	15	11.25	7.5	3.75	0	0
Women	Forward	Set Piece	30	22.5	15	7.5	30	30
Women	Forward	Match-play	58	58	58	58	58	58
Women	Back	Non-Contact	50	63.75	77.5	91.25	50	65

Women	Back	Controlled Contact	40	30	20	10	55	40
Women	Back	Full Contact	15	11.25	7.5	3.75	0	0
Women	Back	Set Piece	0	0	0	0	0	0
Women	Back	Match-play	74	74	74	74	74	74

N.B.: $WRCLG_{current}$: contact durations at current World Rugby contact load guidelines; $WRCLG_{-25\%}$: contact durations reduced by a quarter from current guidelines and replaced with non-contact training; $WRCLG_{-50\%}$: contact durations reduced by half from current guidelines and replaced with non-contact training; $WRCLG_{-75\%}$: contact durations reduced by three quarters from current guidelines and replaced with non-contact training; $WRCLG_{FC-CC}$: full contact training replaced with controlled contact training; $WRCLG_{FC-NC}$: full contact training replaced with non-contact training.

Supplementary Table 2: Simulations of the effect of decreasing contact training duration beyond current World Rugby contact load guidelines on overall in-season *all recorded* HAE exposure in men's rugby. Brackets represent 95% reference ranges for players. Overall HAE exposure provided, which may contain small differences from the median values highlighted in the manuscript.

Matches	$WRCLG_{current}$	$WRCLG_{-25\%}$	$WRCLG_{-50\%}$	$WRCLG_{-75\%}$	$WRCLG_{FC-CC}$	$WRCLG_{FC-NC}$
**Forwards**						
30	1130 (498-2592)	1112 (488-2483)	1077 (478-2470)	1029 (450-2339)	1082 (475-2430)	1065 (476-2373)
25	995 (438-2277)	973 (428-2205)	949 (415-2143)	905 (401-2012)	957 (427-2177)	932 (417-2125)
20	878 (390-2004)	861 (376-1964)	826 (363-1882)	784 (341-1798)	829 (359-1875)	809 (356-1796)
15	748 (332-1722)	733 (320-1651)	700 (307-1580)	656 (289-1487)	692 (307-1576)	675 (300-1543)
10	619 (275-1427)	608 (262-1376)	570 (251-1295)	526 (228-1202)	568 (252-1270)	558 (243-1267)
5	500 (217-1137)	472 (204-1089)	440 (190-996)	401 (178-903)	439 (192-998)	429 (186-986)
**Backs**						
30	733 (323-1643)	724 (319-1604)	714 (312-1594)	690 (309-1579)	663 (291-1488)	661 (293-1498)
25	635 (276-1458)	624 (275-1460)	615 (270-1406)	605 (261-1357)	567 (245-1276)	564 (247-1264)
20	538 (238-1216)	531 (230-1229)	523 (229-1211)	507 (218-1134)	479 (212-1095)	472 (204-1054)
15	444 (192-1024)	439 (191-993)	427 (185-973)	416 (179-945)	378 (166-874)	380 (165-879)
10	355 (153-798)	346 (149-792)	336 (145-769)	320 (139-731)	292 (125-673)	289 (124-657)
5	259 (112-601)	254 (110-584)	243 (104-554)	227 (98-518)	196 (82-454)	194 (83-445)

N.B.: $WRCLG_{current}$: contact durations at current World Rugby contact load guidelines; $WRCLG_{-25\%}$: contact durations reduced by a quarter from current guidelines and replaced with non-contact training; $WRCLG_{-50\%}$: contact durations reduced by half from current guidelines and replaced with non-contact training; $WRCLG_{-75\%}$: contact durations reduced by three quarters from current guidelines and replaced with non-contact training; $WRCLG_{FC-CC}$: full contact training replaced with controlled contact training; $WRCLG_{FC-NC}$: full contact training replaced with non-contact training.

Supplementary Table 3: Simulations of the effect of decreasing contact training duration beyond current World Rugby contact load guidelines on overall in-season HAE >10*g* exposure in men's rugby. Brackets represent 95% reference ranges for players. Overall HAE exposure provided, which may contain small differences from the median values highlighted in the manuscript.

Matches	$WRCLG_{current}$	$WRCLG_{-25\%}$	$WRCLG_{-50\%}$	$WRCLG_{-75\%}$	$WRCLG_{FC-CC}$	$WRCLG_{FC-NC}$
**Forwards**						
30	562 (229-1373)	550 (221-1349)	540 (221-1296)	515 (207-1259)	538 (219-1296)	533 (212-1278)
25	491 (200-1200)	483 (200-1179)	466 (194-1132)	446 (182-1087)	470 (188-1150)	462 (189-1119)
20	425 (171-1025)	414 (167-1012)	400 (161-992)	379 (157-930)	404 (163-1009)	391 (157-958)
15	357 (142-880)	348 (140-858)	336 (136-812)	311 (126-764)	331 (134-807)	325 (133-778)
10	289 (118-711)	279 (111-680)	265 (107-658)	246 (100-614)	267 (105-645)	259 (106-636)
5	222 (90-555)	216 (85-514)	199 (79-483)	181 (72-440)	197 (77-448)	189 (75-471)
**Backs**						
30	329 (134-808)	328 (134-801)	326 (128-809)	318 (127-773)	308 (127-734)	308 (122-758)
25	287 (115-700)	282 (114-690)	281 (112-678)	271 (110-659)	262 (104-640)	260 (107-637)
20	240 (94-594)	238 (95-594)	233 (94-561)	223 (93-547)	217 (87-526)	216 (86-532)
15	193 (77-478)	192 (78-464)	187 (74-461)	182 (72-445)	171 (69-417)	169 (69-417)
10	147 (59-363)	146 (59-360)	141 (57-339)	136 (54-332)	126 (49-309)	125 (48-310)
5	103 (40-252)	100 (39-250)	97 (38-231)	91 (35-227)	81 (31-197)	80 (31-191)

N.B.: $WRCLG_{current}$: contact durations at current World Rugby contact load guidelines; $WRCLG_{-25\%}$: contact durations reduced by a quarter from current guidelines and replaced with non-contact training; $WRCLG_{-50\%}$: contact durations reduced by half from current guidelines and replaced with non-contact training; $WRCLG_{-75\%}$: contact durations reduced by three quarters from current guidelines and replaced with non-contact training; $WRCLG_{FC-CC}$: full contact training replaced with controlled contact training; $WRCLG_{FC-NC}$: full contact training replaced with non-contact training.

Supplementary Table 4: Simulations of the effect of decreasing contact training duration beyond current World Rugby contact load guidelines on overall in-season HAE >25*g* exposure in men's rugby. Brackets represent 95% reference ranges for players. Overall HAE exposure provided, which may contain small differences from the median values highlighted in the manuscript.

Matches	$WRCLG_{current}$	$WRCLG_{-25\%}$	$WRCLG_{-50\%}$	$WRCLG_{-75\%}$	$WRCLG_{FC-CC}$	$WRCLG_{FC-NC}$
**Forwards**						
30	114 (38-340)	113 (37-328)	111 (36-323)	106 (36-308)	110 (35-314)	108 (36-316)
25	99 (32-296)	96 (31-294)	95 (30-280)	91 (30-267)	94 (31-276)	93 (30-280)
20	84 (27-253)	82 (26-246)	80 (26-231)	76 (23-228)	79 (25-235)	78 (25-228)
15	69 (22-210)	68 (22-201)	64 (20-193)	61 (19-177)	63 (19-187)	63 (20-181)
10	54 (16-165)	52 (16-152)	50 (15-144)	46 (14-139)	48 (15-142)	47 (14-139)
5	39 (11-115)	37 (11-112)	35 (10-102)	31 (9-94)	33 (10-99)	32 (9-97)
**Backs**						
30	69 (21-205)	68 (21-205)	68 (21-203)	68 (21-200)	67 (20-202)	66 (21-196)
25	59 (19-179)	59 (19-170)	58 (18-169)	58 (18-166)	56 (18-166)	56 (17-165)
20	49 (15-146)	48 (15-145)	48 (14-140)	47 (14-139)	46 (14-139)	46 (14-133)
15	39 (12-114)	38 (11-112)	37 (11-111)	37 (11-107)	36 (11-107)	35 (11-106)
10	28 (8-85)	28 (8-84)	27 (8-81)	27 (8-79)	26 (7-76)	25 (7-76)
5	18 (5-55)	18 (4-53)	17 (4-53)	16 (4-49)	15 (4-47)	15 (3-46)

N.B.: $WRCLG_{current}$: contact durations at current World Rugby contact load guidelines; $WRCLG_{-25\%}$: contact durations reduced by a quarter from current guidelines and replaced with non-contact training; $WRCLG_{-50\%}$: contact durations reduced by half from current guidelines and replaced with non-contact training; $WRCLG_{-75\%}$: contact durations reduced by three quarters from current guidelines and replaced with non-contact training; $WRCLG_{FC-CC}$: full contact training replaced with controlled contact training; $WRCLG_{FC-NC}$: full contact training replaced with non-contact training.

Supplementary Table 5: Simulations of the effect of decreasing contact training duration beyond current World Rugby contact load guidelines on overall in-season HAE >1000 rad/s^2 exposure in men's rugby. Brackets represent 95% reference ranges for players. Overall HAE exposure provided, which may contain small differences from the median values highlighted in the manuscript.

Matches	$WRCLG_{current}$	$WRCLG_{-25\%}$	$WRCLG_{-50\%}$	$WRCLG_{-75\%}$	$WRCLG_{FC-CC}$	$WRCLG_{FC-NC}$
**Forwards**						
30	370 (149-872)	363 (149-883)	358 (145-875)	359 (144-871)	367 (149-900)	146 (363-877)
25	312 (125-763)	305 (123-755)	304 (127-747)	300 (123-743)	311 (128-764)	309 (126-759)
20	257 (105-639)	254 (102-614)	249 (101-613)	251 (100-608)	260 (106-627)	257 (104-634)
15	204 (82-493)	199 (82-486)	197 (79-479)	195 (79-479)	205 (82-505)	203 (81-504)
10	151 (60-371)	148 (57-362)	142 (56-348)	141 (57-344)	150 (59-382)	148 (58-363)
5	98 (38-241)	36 (92-231)	88 (34-217)	87 (33-214)	98 (38-238)	97 (37-237)
**Backs**						
30	248 (98-599)	246 (97-597)	247 (97-599)	246 (101-598)	251 (102-613)	249 (99-609)
25	210 (85-513)	207 (84-502)	208 (84-501)	206 (85-498)	211 (86-511)	209 (82-504)
20	169 (67-412)	165 (67-416)	167 (66-402)	165 (67-410)	173 (70-420)	169 (67-414)
15	129 (51-326)	130 (51-320)	127 (50-312)	126 (50-309)	132 (51-323)	130 (51-315)
10	90 (35-225)	88 (34-216)	86 (33-217)	86 (33-213)	92 (35-225)	90 (35-224)
5	50 (19-127)	49 (17-122)	47 (17-118)	46 (16-116)	52 (20-131)	50 (18-126)

N.B.: $WRCLG_{current}$: contact durations at current World Rugby contact load guidelines; $WRCLG_{-25\%}$: contact durations reduced by a quarter from current guidelines and replaced with non-contact training; $WRCLG_{-50\%}$: contact durations reduced by half from current guidelines and replaced with non-contact training; $WRCLG_{-75\%}$: contact durations reduced by three quarters from current guidelines and replaced with non-contact training; $WRCLG_{FC-CC}$: full contact training replaced with controlled contact training; $WRCLG_{FC-NC}$: full contact training replaced with non-contact training.

Supplementary Table 6: Simulations of the effect of decreasing contact training duration beyond current World Rugby contact load guidelines on overall in-season HAE >2000 rad/s^2 exposure in men's rugby. Brackets represent 95% reference ranges for players. Overall HAE exposure provided, which may contain small differences from the median values highlighted in the manuscript.

Matches	$WRCLG_{current}$	$WRCLG_{-25\%}$	$WRCLG_{-50\%}$	$WRCLG_{-75\%}$	$WRCLG_{FC-CC}$	$WRCLG_{FC-NC}$
**Forwards**						
30	90 (30-259)	89 (29-257)	86 (29-248)	83 (28-238)	85 (28-247)	84 (28-239)
25	78 (25-227)	76 (25-221)	74 (24-220)	72 (24-210)	73 (24-215)	73 (24-208)
20	66 (21-193)	64 (21-188)	63 (20-177)	59 (19-175)	62 (20-178)	60 (19-175)
15	54 (17-154)	52 (16-152)	51 (16-150)	48 (15-140)	50 (16-145)	48 (16-142)
10	43 (13-124)	41 (13-122)	38 (12-113)	36 (11-107)	38 (12-111)	37 (11-107)
5	30 (9-91)	29 (8-86)	27 (8-81)	25 (7-72)	26 (8-77)	25 (7-76)
**Backs**						
30	68 (22-194)	67 (22-193)	66 (21-190)	65 (21-185)	65 (21-185)	65 (21-186)
25	57 (18-167)	57 (19-167)	56 (18-169)	56 (18-164)	55 (17-160)	55 (18-163)
20	48 (15-142)	47 (15-134)	46 (15-135)	46 (14-135)	46 (14-129)	45 (14-130)
15	39 (12-111)	37 (11-109)	37 (11-107)	36 (11-103)	36 (11-105)	35 (11-105)
10	29 (8-83)	28 (8-81)	27 (8-76)	26 (8-76)	26 (7-74)	25 (7-74)
5	18 (5-55)	18 (5-53)	17 (5-52)	16 (4-50)	16 (4-46)	16 (4-47)

N.B.: $WRCLG_{current}$: contact durations at current World Rugby contact load guidelines; $WRCLG_{-25\%}$: contact durations reduced by a quarter from current guidelines and replaced with non-contact training; $WRCLG_{-50\%}$: contact durations reduced by half from current guidelines and replaced with non-contact training; $WRCLG_{-75\%}$: contact durations reduced by three quarters from current guidelines and replaced with non-contact training; $WRCLG_{FC-CC}$: full contact training replaced with controlled contact training; $WRCLG_{FC-NC}$: full contact training replaced with non-contact training.

Supplementary Table 7: Simulations of the effect of decreasing contact training duration beyond current World Rugby contact load guidelines on overall in-season *all recorded* HAE exposure in women's rugby. Brackets represent 95% reference ranges for players. Overall HAE exposure provided, which may contain small differences from the median values highlighted in the manuscript.

Matches	$WRCLG_{current}$	$WRCLG_{-25\%}$	$WRCLG_{-50\%}$	$WRCLG_{-75\%}$	$WRCLG_{FC\text{-}CC}$	$WRCLG_{FC\text{-}NC}$
**Forwards**						
30	516 (222-1167)	493 (214-1103)	478 (206-1091)	449 (198-1012)	479 (210-1090)	477 (207-1074)
25	454 (198-1016)	433 (190-980)	415 (178-956)	392 (169-884)	420 (180-966)	417 (182-933)
20	388 (172-876)	371 (163-835)	351 (151-812)	326 (143-750)	358 (156-824)	355 (152-809)
15	328 (143-737)	314 (136-705)	292 (126-661)	269 (117-619)	295 (131-675)	298 (129-664)
10	270 (117-614)	250 (106-563)	232 (98-531)	209 (90-469)	238 (102-536)	232 (101-532)
5	208 (89-478)	191 (81-440)	172 (72-396)	150 (62-339)	176 (76-397)	173 (74-391)
**Backs**						
30	386 (168-881)	384 (168-865)	376 (160-861)	368 (161-823)	362 (158-821)	362 (158-821)
25	335 (149-755)	331 (146-750)	321 (139-734)	312 (138-705)	310 (135-710)	310 (135-710)
20	287 (127-650)	280 (120-639)	274 (119-623)	262 (113-590)	258 (114-596)	258 (114-596)
15	236 (101-540)	230 (98-531)	222 (97-496)	212 (92-481)	210 (89-473)	210 (89-473)
10	186 (79-419)	180 (75-411)	170 (73-382)	160 (69-368)	158 (66-362)	158 (66-362)
5	134 (58-312)	128 (54-296)	119 (51-283)	110 (47-256)	107 (44-248)	107 (44-248)

N.B.: $WRCLG_{current}$: contact durations at current World Rugby contact load guidelines; $WRCLG_{-25\%}$: contact durations reduced by a quarter from current guidelines and replaced with non-contact training; $WRCLG_{-50\%}$: contact durations reduced by half from current guidelines and replaced with non-contact training; $WRCLG_{-75\%}$: contact durations reduced by three quarters from current guidelines and replaced with non-contact training; $WRCLG_{FC\text{-}CC}$: full contact training replaced with controlled contact training; $WRCLG_{FC\text{-}NC}$: full contact training replaced with non-contact training.

Supplementary Table 8: Simulations of the effect of decreasing contact training duration beyond current World Rugby contact load guidelines on overall in-season HAE >10*g* exposure in women's rugby. Brackets represent 95% reference ranges for players. Overall HAE exposure provided, which may contain small differences from the median values highlighted in the manuscript.

Matches	$WRCLG_{current}$	$WRCLG_{-25\%}$	$WRCLG_{-50\%}$	$WRCLG_{-75\%}$	$WRCLG_{FC-CC}$	$WRCLG_{FC-NC}$
**Forwards**						
30	227 (92-554)	220 (86-539)	213 (84-516)	203 (80-496)	213 (87-517)	210 (83-531)
25	196 (77-486)	189 (74-466)	182 (72-441)	174 (71-421)	184 (75-441)	183 (72-445)
20	167 (67-415)	161 (63-396)	152 (61-376)	145 (58-360)	154 (61-384)	154 (61-374)
15	141 (56-336)	132 (52-321)	125 (49-307)	115 (46-275)	125 (49-303)	124 (50-306)
10	111 (43-271)	102 (40-252)	95 (37-234)	87 (33-211)	96 (37-233)	95 (37-235)
5	82 (31-203)	73 (28-180)	66 (25-167)	57 (21-139)	66 (25-163)	65 (24-163)
**Backs**						
30	168 (64-415)	168 (67-405)	164 (67-393)	162 (63-401)	155 (61-373)	156 (62-383)
25	145 (56-354)	144 (57-352)	142 (56-350)	139 (56-335)	132 (52-324)	131 (51-325)
20	123 (49-295)	121 (46-300)	118 (46-294)	116 (46-285)	109 (43-269)	109 (43-274)
15	99 (38-243)	98 (38-244)	97 (37-237)	95 (36-232)	86 (33-214)	87 (33-213)
10	77 (29-188)	77 (30-190)	74 (28-180)	71 (27-174)	63 (24-156)	63 (24-155)
5	54 (20-134)	53 (20-131)	51 (19-127)	48 (18-121)	41 (15-101)	40 (14-101)

N.B.: $WRCLG_{current}$: contact durations at current World Rugby contact load guidelines; $WRCLG_{-25\%}$: contact durations reduced by a quarter from current guidelines and replaced with non-contact training; $WRCLG_{-50\%}$: contact durations reduced by half from current guidelines and replaced with non-contact training; $WRCLG_{-75\%}$: contact durations reduced by three quarters from current guidelines and replaced with non-contact training; $WRCLG_{FC-CC}$: full contact training replaced with controlled contact training; $WRCLG_{FC-NC}$: full contact training replaced with non-contact training.

Supplementary Table 9: Simulations of the effect of decreasing contact training duration beyond current World Rugby contact load guidelines on overall in-season HAE >25*g* exposure in women's rugby. Brackets represent 95% reference ranges for players. Overall HAE exposure provided, which may contain small differences from the median values highlighted in the manuscript.

Matches	$WRCLG_{current}$	$WRCLG_{-25\%}$	$WRCLG_{-50\%}$	$WRCLG_{-75\%}$	$WRCLG_{FC-CC}$	$WRCLG_{FC-NC}$
**Forwards**						
30	39 (11-114)	37 (11-109)	37 (11-110)	36 (11-109)	37 (11-110)	37 (11-107)
25	33 (10-98)	32 (10-97)	31 (9-93)	30 (9-91)	31 (9-93)	31 (9-95)
20	27 (8-82)	27 (7-79)	26 (7-78)	25 (7-77)	26 (7-80)	26 (7-75)
15	22 (6-68)	22 (6-66)	21 (5-62)	20 (5-60)	20 (5-62)	21 (5-63)
10	17 (4-52)	16 (4-50)	16 (4-47)	15 (3-46)	15 (4-46)	15 (4-46)
5	12 (3-37)	11 (2-35)	10 (2-31)	9 (2-29)	10 (2-31)	10 (2-31)
**Backs**						
30	33 (10-101)	34 (10-105)	33 (10-101)	33 (10-101)	31 (9-94)	32 (9-94)
25	29 (8-86)	29 (8-85)	29 (8-83)	28 (8-86)	26 (8-79)	26 (7-81)
20	24 (6-71)	24 (6-71)	24 (6-72)	24 (6-71)	21 (6-65)	22 (6-66)
15	19 (5-56)	19 (5-57)	19 (5-57)	19 (5-58)	17 (4-51)	17 (4-51)
10	14 (3-43)	14 (3-42)	14 (3-42)	14 (3-43)	12 (3-36)	12 (3-36)
5	9 (2-28)	9 (2-27)	9 (2-29)	9 (1-28)	7 (1-22)	7 (1-22)

N.B.: $WRCLG_{current}$: contact durations at current World Rugby contact load guidelines; $WRCLG_{-25\%}$: contact durations reduced by a quarter from current guidelines and replaced with non-contact training; $WRCLG_{-50\%}$: contact durations reduced by half from current guidelines and replaced with non-contact training; $WRCLG_{-75\%}$: contact durations reduced by three quarters from current guidelines and replaced with non-contact training; $WRCLG_{FC-CC}$: full contact training replaced with controlled contact training; $WRCLG_{FC-NC}$: full contact training replaced with non-contact training.

Supplementary Table 10: Simulations of the effect of decreasing contact training duration beyond current World Rugby contact load guidelines on overall in-season HAE >1000 rad/s^2 exposure in women's rugby. Brackets represent 95% reference ranges for players. Overall HAE exposure provided, which may contain small differences from the median values highlighted in the manuscript.

Matches	$WRCLG_{current}$	$WRCLG_{-25\%}$	$WRCLG_{-50\%}$	$WRCLG_{-75\%}$	$WRCLG_{FC\text{-}CC}$	$WRCLG_{FC\text{-}NC}$
**Forwards**						
30	182 (72-440)	179 (72-453)	177 (72-431)	175 (70-419)	178 (70-424)	178 (71-440)
25	160 (64-392)	156 (61-382)	150 (61-370)	148 (59-354)	153 (60-375)	153 (60-377)
20	135 (53-331)	131 (52-318)	127 (51-311)	123 (48-306)	128 (50-307)	128 (51-314)
15	109 (42-266)	106 (41-264)	102 (40-252)	100 (40-245)	104 (40-253)	103 (40-254)
10	85 (33-206)	81 (31-200)	78 (30-195)	74 (29-184)	78 (30-196)	79 (30-194)
5	59 (22-145)	56 (21-139)	53 (20-130)	50 (19-125)	54 (20-134)	55 (20-133)
**Backs**						
30	150 (60-359)	150 (59-373)	149 (59-368)	146 (60-357)	144 (57-353)	143 (56-352)
25	129 (52-314)	129 (51-316)	128 (50-315)	126 (49-308)	123 (49-302)	122 (47-298)
20	107 (42-262)	108 (42-262)	105 (40-258)	104 (41-250)	100 (39-252)	101 (38-247)
15	87 (33-213)	85 (33-207)	85 (33-206)	83 (32-205)	81 (31-194)	79 (30-196)
10	64 (24-158)	64 (25-161)	64 (23-156)	61 (23-152)	58 (22-142)	58 (22-144)
5	44 (16-108)	42 (16-107)	42 (15-105)	41 (15-99)	37 (13-93)	37 (13-91)

N.B.: $WRCLG_{current}$: contact durations at current World Rugby contact load guidelines; $WRCLG_{-25\%}$: contact durations reduced by a quarter from current guidelines and replaced with non-contact training; $WRCLG_{-50\%}$: contact durations reduced by half from current guidelines and replaced with non-contact training; $WRCLG_{-75\%}$: contact durations reduced by three quarters from current guidelines and replaced with non-contact training; $WRCLG_{FC\text{-}CC}$: full contact training replaced with controlled contact training; $WRCLG_{FC\text{-}NC}$: full contact training replaced with non-contact training.

Supplementary Table 11: Simulations of the effect of decreasing contact training duration beyond current World Rugby contact load guidelines on overall in-season HAE >2000 rad/s[2] exposure in women's rugby. Brackets represent 95% reference ranges for players. Overall HAE exposure provided, which may contain small differences from the median values highlighted in the manuscript.

Matches	$WRCLG_{current}$	$WRCLG_{-25\%}$	$WRCLG_{-50\%}$	$WRCLG_{-75\%}$	$WRCLG_{FC-CC}$	$WRCLG_{FC-NC}$
**Forwards**						
30	39 (12-115)	37 (11-109)	35 (10-102)	34 (10-98)	38 (11-110)	37 (12-110)
25	35 (10-99)	32 (9-95)	30 (8-87)	28 (8-84)	32 (10-94)	33 (9-93)
20	28 (9-82)	27 (8-77)	25 (7-73)	24 (7-68)	27 (8-79)	27 (8-80)
15	23 (7-68)	21 (6-61)	20 (5-56)	18 (5-54)	22 (6-64)	22 (6-65)
10	18 (5-54)	16 (4-50)	15 (3-44)	13 (3-39)	17 (4-50)	17 (4-50)
5	13 (3-38)	11 (2-34)	2 (9-28)	8 (1-24)	12 (3-35)	11 (3-35)
**Backs**						
30	35 (11-104)	35 (11-103)	35 (11-102)	11 (35-103)	33 (10-98)	33 (10-98)
25	30 (9-89)	30 (9-90)	30 (9-89)	31 (9-88)	28 (8-82)	29 (8-85)
20	26 (7-77)	25 (7-74)	25 (7-76)	25 (7-75)	23 (7-68)	24 (7-69)
15	20 (6-61)	20 (6-60)	20 (5-61)	20 (5-59)	19 (5-54)	18 (5-55)
10	15 (4-46)	15 (4-47)	15 (4-45)	15 (4-45)	13 (3-40)	14 (3-41)
5	2 (10-31)	10 (2-31)	10 (2-31)	10 (2-31)	8 (2-26)	8 (2-26)

N.B.: $WRCLG_{current}$: contact durations at current World Rugby contact load guidelines; $WRCLG_{-25\%}$: contact durations reduced by a quarter from current guidelines and replaced with non-contact training; $WRCLG_{-50\%}$: contact durations reduced by half from current guidelines and replaced with non-contact training; $WRCLG_{-75\%}$: contact durations reduced by three quarters from current guidelines and replaced with non-contact training; $WRCLG_{FC-CC}$: full contact training replaced with controlled contact training; $WRCLG_{FC-NC}$: full contact training replaced with non-contact training.
